# Book Review: Ranking: The Unwritten Rules of the Social Game We All Play

**DOI:** 10.3389/fpsyg.2021.605886

**Published:** 2021-03-29

**Authors:** Basabdatta Sen Bhattacharya

**Affiliations:** Department of Computer Science and Information Systems, Birla Institute of Technology and Science (BITS) Pilani, Goa, India

**Keywords:** ranking, rating, lists, hierarchy, comparison, ranking games, reputation, cognitive bias

There is an ever-increasing hum-and-haw in our daily lives due to the multitude of choices at our disposal. This has led to the establishment of lists, scoring, and ranking systems on the world wide web. Ranked lists thrive primarily because they provide a sense of objective measure that appeals to the human brain, which is wired to work best when there is a reference point.

Some rankings are based on subjective experiences of humans, for example, hotel reviews on Tripadvisor. Other rankings are calculated by machines that execute algorithms which are written by humans. This begs the question: is ranking objective or subjective? Is ranking a feature of modern times? Perhaps a by-product of Google's dominance of the internet? Or, is this a natural product of the evolutionary neuronal wiring that guides our social behavior?

Peter Erdi's book *Ranking: The Unwritten Rules of the Social Games We All Play* provides a way of entering this fascinating world. Erdi's account is a stimulating story of the origins of ranking in our society and its impact on the social behavior of humans and animals. Anecdotal narratives, incidents, and experiences are drawn from the author's life, to explain the hard and complex concepts that underlie ranking and rating, which is a consistent feature of the book.

In the Prolog the author paints a nostalgic picture of his childhood, when the love of soccer served as the first lesson on how post-war socio-economic settings impact simple rating exercises for school going boys. He describes how ratings of soccer players by journalists were based on their subjective take on a player's performance or even personality, an example of the “*illusion of objectivity”* that informs objective ranking. The author discusses two other means of objective ranking, including the social dynamics underlying election manipulation and “*self-appointed rankers.”*

From Chapter 2 onwards, the author starts to dive deeper into the topic. Comparison is second nature to people as cognitive and social beings. Humans rank themselves by comparing themselves with neighbors, friends, colleagues, and others. To demonstrate such “extrinsic driven self-ranking,” the author notes the following: “*I bought a six-year-old Trabant in my mid-30s as my first car.”* Loaded with humor (and one of my favorite sentences in the book), it is a demonstration of how one's social rank is often associated with their financial assets. This reference is followed by a delightfully sarcastic exchange between a donkey and a Trabant. Underlying this story, weaving in humor and anecdotes, is a demonstration of how mathematics and sports are the only two fields that come close to handling ranked lists in the purest form possible.

The author's eye for details stands out. Readers will learn about motor cars in post-world-war Eastern Europe, whilst at the same time becoming aware of the Erdos Number, a measure that mathematicians and scientists use to rank themselves, and the Elo number, which was originally used to rank chess players. The most fun part of this section is the story of Rudyard Kipling's novel *Kim* when he was being trained to remember “*long lists”* — enthusiastic readers will be tempted to give it a go.

As Erdi writes, “*while we are not very good at memorizing long lists, lists still help us process sensory information. In addition, reading listicles can give us the impression that we have gained complete knowledge about a certain topic*.” [p. 211].

Chapter 3 discusses the story of a plethora of ranking mechanisms — from the social ranking implied by the Japanese bowing hierarchy and Roman Toga colors, to Network Society, a laterally connected (as opposed to vertical i.e., hierarchical) society that may have more than one “leader” depending on what measure is used i.e., what is the problem at hand.

When one talks about ranking, can the multinational technology company Google go unmentioned? Chapter 4 introduces and discusses how and why Google's page algorithm came to top the “*ranked list of Search Engines.”* Note that we are now ranking the rankers; more specifically, we are “*Ranking the Ranking of the Rankers.”* The author draws attention to this never-ending loop — “*when you see a list of colleges or countries based on any criteria, nobody will tell you that the ranked list is produced by some random algorithms.”* By changing just one parameter in the ranking algorithm, the output, i.e., the order of the ranked list changes.

Chapters 4 and 5 discuss the many “*sources of cognitive bias and its effect on our thoughts and behaviors. Because we are presented with an abundance of information and limited capacity for making decisions, we use mental shortcuts or heuristics to help us act on the information presented by the world around us. One such heuristic that we previously mentioned is satisficing, seeking a “good-enough” decision rather than a perfect one. But there are many heuristics we unconsciously use in our day-to-day lives as a means of compensating for our cognitive shortcomings. This points to our fascination with lists”* [p. 127].

Chapters 6 to 8 take us on a joy ride with the different facets of the “*Ranking games”* that we as a society play every day, including worldwide university rankings, fake reviews on Tripadvisor, and a nuanced analysis (in context) of the recent controversies concerning the Oscar awards ceremonies. The discussion on quantifying artistic success is noteworthy.

As Erdi observes, whether we “*like it or not, ranking is with us. It is not a magic bullet that produces order out of chaos, but it is not the product of some random procedure (Figure 9.1)”* [p. 207]. As a reputed scientist and a teacher in complex systems, it is perhaps not surprising that Erdi deftly handles the extremely vast and complex theories of ranking and rating, and how they impact our everyday lives. This serious issue is well-narrated with engrossing anecdotal examples, and a generous dose of (subtle) humor and will resonate with professionals and scholars from all walks of life, a must-read.

## Author Contributions

The author confirms being the sole contributor of this work and has approved it for publication.

## Conflict of Interest

The author declares that the research was conducted in the absence of any commercial or financial relationships that could be construed as a potential conflict of interest.

